# Gross-total resection in optic nerve sheath meningiomas: minimally invasive and cosmetic pleasing

**DOI:** 10.1007/s00417-024-06447-4

**Published:** 2024-03-19

**Authors:** Qin Dai, Xinyu Li, Yenan Fang, Bingyan Shen, Jinfei Wei, Qiqi Xie, Wencan Wu, Min Wang

**Affiliations:** 1 National Clinical Research Center for Ocular Diseases, Eye Hospital, Wenzhou Medical University, Wenzhou, 325027 China; 2 State Key Laboratory of Ophthalmology, Optometry and Visual Science, Eye Hospital, Wenzhou Medical University, Wenzhou, 325027 China

**Keywords:** Optic nerve sheath meningiomas, Endoscopic transnasal surgery, Optic nerve transection, Lateral orbitotomy approach, Gross-total resection

## Abstract

**Purpose:**

The optic nerve sheath meningioma (ONSM) is one of the most challenging tumors in orbital surgery. From the perspective of mental health and patient needs, we analyzed the necessity and importance of the endoscopic transnasal approach (ETA) combined with optic nerve transection (ONT) in gross-total resection (GTR) in ONSM patients with residual vision and aim to broaden the use of ONT for specific people.

**Methods:**

The authors included patients with ONSMs who were treated between 2014 and 2022. We divided those cases into two groups named ETA group and lateral orbitotomy approach (LOA) group. We present the application of ETA and analyze the preoperative indication of the ONT and compared the advantages and disadvantages between ETA and LOA. The degree of tumor resection was based on imaging and surgical evaluation.

**Results:**

A total of 23 patients with ONSM were included. Sixteen patients underwent ETA, and seven underwent LOA. Among ETA cases, GTR was achieved in 14 patients with ONT and most patients maintained normal eye movement function (75%) and morphology (93.75%). In the ETA group, 14 patients experienced vision loss, while two other patients saw improvements in vision. And proptosis was alleviated (5.20 ± 2.34 vs 0.27 ± 0.46, *p* < 0.0001). Six patients with blindness and proptosis of the LOA group resulted in GTR with ONT and ophthalmectomy. Although intracranial extension and recurrence included no cases in the two groups, a significant psychological gap was presented due to cosmetic problems.

**Conclusions:**

Under the premise of reducing damage and improving aesthetics, the selection of ETA combined with ONT to gross-total resect ONSMs successfully provides a minimally invasive access with acceptable complications. As an important adjunct to GTR in the surgical treatment of ONSM, the scope of ONT application should be expanded to relieve the patient’s psychological burden.

**Supplementary Information:**

The online version contains supplementary material available at 10.1007/s00417-024-06447-4.


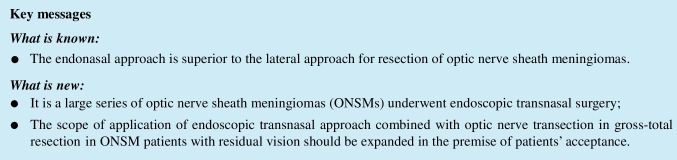


## Introduction

Optic nerve sheath meningiomas (ONSMs) are a rare type of tumor that make up approximately one-third of all intrinsic tumors originating from the optic nerve [1]. These tumors typically cause progressive optic nerve dysfunction, leading to a range of visual deficits. In the past, external craniofacial approaches such as large craniotomy, orbital unroofing, and lateral orbitotomy are commonly utilized to gross-total or subtotal resect tumors [2]. Those traditional surgical approaches to ONSMs require craniotomy and extensive bone removal to access the tumor, which can increase the risk of complications and leave significant cosmetic defects. As the need for less invasive techniques has grown in recent years, endoscopic transnasal approach (ETA) has emerged as a promising alternative for treating ONSMs. Before the ETA is technologically mature in our institution, we tend to use the lateral orbitotomy approach (LOA) for the surgical excision of ONSMs. In comparison to the LOA [3], ETA provides wider access to the optic canal and orbital apex, panoramic visualization, and a reduced risk of damaging normal structures [4, 5]. These advantages have a positive impact on treatment outcomes, lowering the likelihood of recurrence and reducing the invasiveness of the procedure [6]. However, intraorbital tumors that involve the optic nerve (ON) are relatively rare, requiring surgical intervention only in symptomatic cases. As a result, there is very little literature available documenting the experience of endoscopic transnasal resection methods, especially in gross-total resection (GTR) of ONSMs. The characteristic of meningiomas encasing the optic nerve makes GTR particularly challenging without compromising the integrity of the optic nerve [7, 8]. Therefore, to avoid tumor recurrence and intracranial invasion, optic nerve transection (ONT) was inevitable for GTR.

In this paper, we present our experience managing ONSMs in 16 patients using ETA and in seven patients treated with LOA. We investigated the necessity and suitability of ETA and ONT in ONSM with residual vision and proposed a set of comprehensive new perspectives to evaluate the need for ONT. Ultimately, we aim to expand the use of ONT for specific groups of people. We also compared the advantages and disadvantages of ETA and LOA. To the best of our knowledge, this is one of the largest endoscopic surgical series of ONSMs and represents the widest documented experience with ETA in tumor resection.

## Methods

### Patient characteristics

Between February 2014 and August 2022, authors retrospectively reviewed the medical files and imaging studies of patients with optic nerve sheath meningiomas at the Eye Hospital, School of Ophthalmology & Optometry, Wenzhou Medical University, under ethics committee approval. Twenty-three patients with ONSM whose diagnoses were based by radiological, pathological, and immunohistochemical findings. Meningiomas complicating with craniofacial fibrous dysplasia or extending into the optic canal from the intracranial cavity were excluded.

In this study, a total of 23 patients were included, none of whom had undergone radiation therapy before. Based on the surgical approach used, the 23 cases were divided into two groups: the ETA group (*n* = 16) and the LOA group (*n* = 7). In the ETA group, 14 received ETA combined with ONT. The remaining two patients received targeted approaches including ETA and endoscopic optic nerve decompression (EOND). Among seven patients in the LOA group, six cases with ONT, and five underwent ophthalmectomy (OM). The transconjunctival approach was a valuable auxiliary approach in our series.

The ophthalmological examination consisted of testing the patients’ visual acuity (Snellen notation), fundoscopy, pattern visual evoked potential (PVEP) test, and Humphrey static threshold automated perimetry for visual field defects. According to the Glaucoma Staging System (GSS) [9], we applied this classification system in those patients. Patients were contacted by phone to obtain recent examination data. Follow-up data were determined by the last date of postoperative computed tomographic (CT) and/or magnetic resonance imaging (MRI) examinations. And none showed signs of complications and recurrence. The mean hospitalization time in the ETA group was 6.5 (range 5–12) days and was 5.3 (range 4–6) days in the LOA group.

Preoperative CT or MRI scans were performed on all patients, but only 15 for the ETA group and six for the LOA group had films available for the retrospective study. The maximum diameter is determined by radiological findings. High-resolution CT showed tumor locations relative to the cerebrum and defined three types: intraorbital (IO), optic canal (OC), and intracranial (IC) space. MRI was done in three planes to analyze the relation to vascular, optic nerve and neighboring structures. Based on Schick et al. [10], we matched the ONSM to the corresponding type. The extent of resection was evaluated by several dedicated oculoplastic surgeons and based on imaging and surgical evaluation. Gross-total resection (GTR), near-total resection (NTR), and subtotal resection (STR) were defined respectively as 100%, 90–99%, and < 90% of the tumor were resected.

### Surgical consideration

**Fig. 1:**
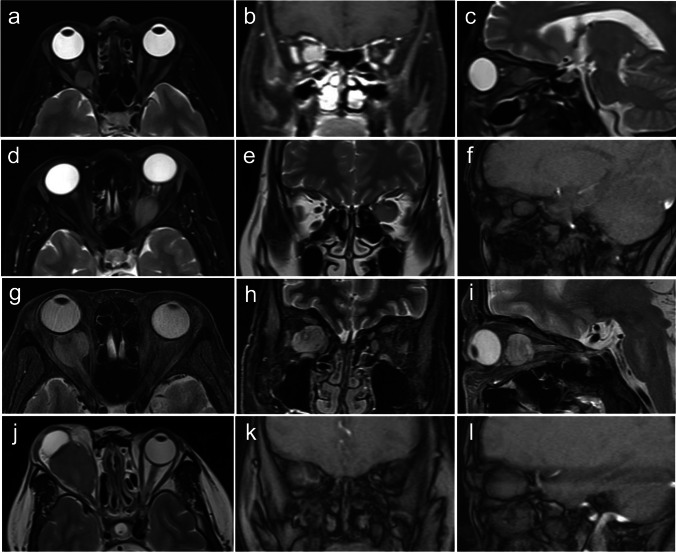
Four cases of intraorbital optic nerve sheath meningiomas (ONSMs). Preoperative magnetic resonance imaging (MRI) showed the location of tumors and surgical considerations. **a**–**c** Case 6 MRI with gadolinium (Gd) enhancement which underwent endoscopic transnasal approach (ETA) only. **a** Axial T2-weighted MR image; **b** coronal T1-weighted MR image; **c** sagittal T2-weighted MR image. **d**–**f**, **g**–**i**, and **j**–**l** Case 20, case 4, and case 24 MR images with/without Gd enhancement which underwent ETA combined with transconjunctival approach (TCA). **d** Axial T2-weighted MR image with Gd enhancement; **e**, **f** coronal and sagittal T2-weighted MR image; **g**–**i** axial, coronal and sagittal T2-weighted MR image; **j** axial T2-weighted MR image; **k**, **l** coronal and sagittal T1-weighted MR image

The main goal of surgery was GTR of the tumor in most patients. In two patients, the surgeries were performed for decompression and diagnostic purposes. Reasons for planned ONT and GTR of tumor included middle-aged and elderly patients with advanced preoperative vision loss, disfiguring proptosis, the tendency of intracranial extension, and patient’s strong desire for surgical total excision. Partial resection of the mass was performed with the patient requesting optic nerve preservation. ETA alone was suitable for a kind of small tumor located posterior to the optic nerve (Fig. 1a–c). And indications for combined ETA with transconjunctival approach (TCA) included large tumor volume and the lesions were accompanied by more than two-thirds of the length of the optic nerve (Fig. 1d–l). TCA and ONT were designed to assist ETA in the complete transection of the tumors and provide a path to remove the lesions. In those seven patients who underwent LOA, meningioma almost filled the orbital space and caused severe compression of the optic nerve. The LOA extended beyond the borders of the tumor to access the orbit apex and remove the involved optic nerve and other contents.

### Surgical technique

#### ETA procedure

**Fig. 2:**
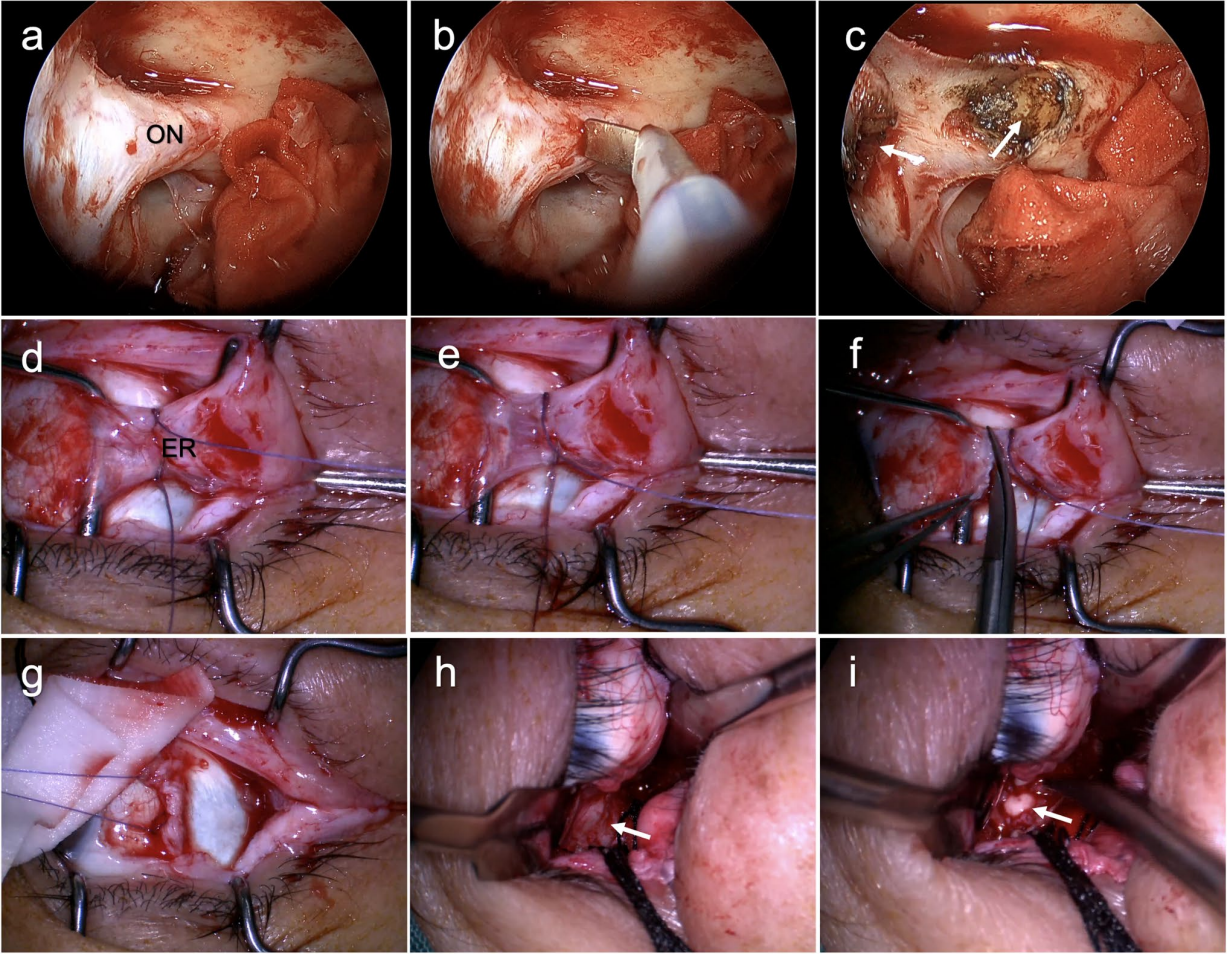
Intraoperative views of the endoscopic transnasal approach and transconjunctival approach for ONSM resection. **a** Complete visualization of the right optic canal; **b**, **c** using an electric knife to dissect the optic nerve, as indicated by the white arrow, showing the severed end of the optic nerve; **d** following the incision of the conjunctiva, the muscle was detached from the eyeball based on its anatomical position; **e** clamping the lateral rectus muscle in place facilitates cutting; **f** on the lateral side, detached the lateral rectus muscle; **g** After incision of the conjunctiva, the muscle was detached from the bulb according to the position of the muscle; **h**, **i** gently separated the tissues behind the globe, located the optic nerve, cut it, and then gently peeled away the tumor from the periorbita. ON, optic nerve; ER, external rectus

After inducing general anesthesia, uncinate process resection was performed to expose the ethmoid bulla. Subsequently, patients underwent endoscopic transnasal unilateral whole group ethmoidectomy using a 4.0-mm, 0-degree endoscope (Karl Storz, Tuttlingen, Germany) and the sphenoid sinus and maxillary sinus were fully exposed; hence, a part of the maxilla was removed. Resecting the unilateral lamina papyracea to the orbital tip to explore the optic nerve at the orbital apex (Fig. 2a). The optic nerve was severed with an electrosurgical cutting system (ICC 80, ERBE, Germany) (Fig. 2b–c). When the mass is small or just a flat thin layer around the optic nerve, ENOD or tumor biopsy can be performed without optic nerve injury. For the most orbital optic nerve tumors, we approached the tumor from a temporal conjunctiva incision (Fig. 2d–i). Following a relaxing superomedial incision in the conjunctiva, the lateral rectus muscle was isolated and secured at its insertion into the globe using a double-armed suture. After separating the lateral rectus muscle from the intermuscular septa (Fig. 2d–e), and cutting the distal ligaments from their insertion position (Fig. 2f), dissecting tissues such as retrobulbar fat behind the eyeball (Fig. 2g), exposing the retrobulbar optic nerve (Fig. 2h), and severing the optic nerve (Fig. 2i), the tumor was separated and completely removed via the conjunctival approach. Online Resource 1 demonstrates the surgical procedure of transecting the optic nerve via the endoscopic transnasal approach and transecting the retrobulbar optic nerve via the conjunctival approach. Due to the absence of recordings for other surgical steps, they are not depicted in the video. During the surgical procedure, hemostasis was achieved by applying pressure with gauze or surgical sponges, or by using an electrocautery to heat and cauterize tissue, sealing off blood vessels, and preventing bleeding.

#### LOA procedure

The lateral orbitotomy approach used an S-shaped brow or horizontal cantholysis skin incision to expose the periosteum of the lateral orbital rim, and dissection began along the superior temporal quadrant with a stripper. Subperiosteal dissection was performed medially to mobilize and free the entire orbital contents from the orbital wall. From the lateral orbit, the orbital apex was cauterized and orbital contents were removed carefully. To select the most appropriate anatomical corridor to orbital tumor, TCA was combined with LOA for patients who need. To completely excise the tumor, five patients underwent enucleation surgery.

### Statistical analysis

Statistical analyses were performed using GraphPad Prism version 9.0. A two-tailed unpaired *t* test was used to compare two independent groups. The results were considered significant at *p* < 0.05 and are reported as the mean ± SD.

## Results

### Signs and symptoms

The patient series in both groups had a high female predominance (86.94%) and a mean age of 49.73 years at diagnosis, consistent with most reports in the literature. Symptomatic duration ranged from 1 to 48 months, and the mean follow-up was 18.61 months. Patients who were followed up for more than 2 years postoperatively did not experience serious injuries or illnesses during the follow-up period. Clinical manifestations included progressive visual decline (*n* = 15), proptosis (*n* = 14), dizziness (*n* = 1), and headache (*n* = 2). In 65.22% of patients, visual performance at presentation was poor (Snellen equivalent < 0.1 to 0), and in 30.43% of patients, it was fair (< 0.5 to ≥ 0.1). Only one (4.35%) patient had good (≥ 0.5) preoperative vision. The relative degree of exophthalmos of the eyeball ranged from 2 to 9 mm. Other clinical symptoms were also consistent with the disease characteristics, such as relative afferent pupillary dysfunction (65.22%), pale optic papilla (52.17%), and optic disc edema (13.04%). Visual field examination was available for 16 patients, and most cases presented GSS stage 5, indicating significant visual field impairment. Pattern visual evoked potential (PVEP) examination results were available for 15 patients, and all showed delayed P100 peak amplitude, indicating visual transmission dysfunction. In terms of pathology, the most common histopathologic pattern was meningothelial. Three cases were classified as psammomatous, and the remaining two tumors were microcystic and mixed meningothelial and angiomatous, respectively. And four patients had recurrent lesions (Table 1).

**Table 1:** Clinical characteristics of 23 patients

Parameter	Number (%)
Sex (male:female)	3 (13.04):20 (86.94)
Ages (mean ± SD, years)	49.73 ± 10.88
Affected side (right:left)	10 (43.48):13 (56.52)
Symptomatic duration (mean ± SD, months)	15.26 ± 12.89
Follow-up (mean ± SD, months)	18.61 ± 24.13
Initial symptoms	
Blurred vision	15 (65.22)
Proptosis	15 (65.22)
Preoperative proptosis (mean ± SD, mm)	5.20 ± 2.34
Postoperative proptosis (mean ± SD, mm)	0.27 ± 0.46
Headache	2 (8.70)
Dizziness	1 (4.35)
Visual performance	
Poor	15 (65.22)
Fair	7 (30.43)
Good	1 (4.35)
Examination	
Relative afferent pupillary defect	15 (65.22)
Pale optic papilla	12 (52.17)
Optic disc edema	3 (13.04)
Computerized perimetry examination	16 (69.57)
Glaucoma Staging System	
Stage 0	0
Stage 1	1
Stage 2	0
Stage 3	1
Stage 4	3
Stage 5	11
PVEP P100	15 (65.22)
Delayed peak amplitude	15
Pathology	
Meningothelial	18 (78.26)
Psammomatous	3 (13.04)
Microcystic	1 (4.35)
Mixed meningothelial and angiomatous	1 (4.35)
Recurrent	4 (17.39)

*PVEP* Pattern visual evoked potential

Preoperative visual examination results of 14 patients who underwent ONT are shown in Table 2. Compared with contralateral eyes, affected eyes demonstrated a significant decline in vision-related indicators, with a mean Snellen visual acuity of 0.16. Although most of these 14 patients have residual vision before surgery, data from visual fields testing was available for 12 ONT cases, presenting as numerical data, including visual field index (VFI), mean deviation (MD), and pattern standard deviation (PSD), showing absolute incomplete visual field. Additionally, PVEP was available for 10 ONT cases and showed a significant delay (*p* < 0.05, *t* = 0.284), indicating problems with visual field and optic nerve conduction.

**Table 2:** Preoperative visual examination results of 14 patients with ONT in ETA group

	BCVA (Snellen notation)	Visual field			PVEP P100 (ms)
		VFI (%)	MD (dB)	PSD (dB)	
Affected eye	0.16 ± 0.19	24.33 ± 32.78	− 25.47 ± 9.02	5.55 ± 3.92	125.60 ± 20.80
Contralateral eye	0.70 ± 0.29	96.62 ± 4.13	− 2.31 ± 2.56	3.11 ± 2.05	105.90 ± 13.49
*t* value	5.537	7.866	8.516	1.888	0.284
*p* value	< 0.0001	< 0.0001	< 0.0001	0.0714	0.0284

*BCVA* Best-corrected visual acuity; *VFI* Visual field index; *MD* Mean deviation; *PSD* Pattern standard deviation; *PVEP* Pattern visual evoked potential

### Clinical outcome

None of the patients in this series experienced a worsening of their initial symptoms after undergoing ETA or LOA. Furthermore, all patients experienced postoperative improvement of preoperative proptosis. Among patients who presented with preoperative visual symptoms, two showed slight improvement after undergoing ETA (case 12) and EOND (case 14), respectively. This result may be related to the involvement of optic nerve decompression and a relatively short symptomatic and postoperative duration.

Details of the 23 included patients and their surgical outcomes are described in Table 3. According to the type of tumor, types Ia, Ib, Ic, IIa, and IIIa were involved in one, 11, four, three, and two cases, respectively. Tumor maximum diameter varied from 3 to 40 mm. There were twenty cases involving the optic nerve, which accounted for over two-thirds of the total. The identical eye globe relative proptosis symptoms of all patients showed improvement (5.20 ± 2.34 vs 0.27 ± 0.46, *p* < 0.0001).

**Table 3:** Summary of data in 23 patients with ONSMs underwent ETA or LOA

Group	Case No	Sex/age (yrs)	Tumor type*	Max diameter (mm)	Preop proptosis (mm)	Postop proptosis (mm)	Preop vision	Postop vision	Tumor location	Surgical approach†	EOR	FU (mos)
ETA	17	M/43	Ia	13	3	0	0.4	0	IO	1, t	GTR	27
	11	F/52	Ib	24	5	0	0.25	0	IO	2, t	GTR	34
	18	F/34	Ib	11	3	0	0	0	IO	2, t, OM	GTR	21
	23	F/43	Ib	10	NA	NA	0.008	0	IO + OC	2, t	GTR	5
	24	F/40	Ib	38	7	1	0.02	0	IO	2, t	GTR	0
	27	F/36	Ib	34	9	1	0.4	0	IO	2, t	GTR	1
	4	F/64	Ib	22	3	0	0.001	0	IO	2, t	GTR	6
	6	F/55	Ic	13	NA	NA	0.25	0	IO	1, t	GTR	0
	20	F/42	Ic	19	5	0	0.6	0	IO	2, t	GTR	12
	22	F/54	Ic	15	NA	NA	0.25	0	IO	2, t	GTR	19
	26	F/53	Ic	12	2	0	0.1	0	IO	2, t	GTR	8
	14	M/46	IIa	3	NA	NA	0.02	0.12	IO + OC	3	-	36
	21	F/45	IIa	29	NA	NA	0	0	IO + OC	2, t	GTR	18
	28	F/55	IIa	29	NA	NA	0.01	0	IO + OC	2, t	GTR	62
	29	F/51	IIIa	31	6	1	0	0	IO + OC + IC	2, t	GTR	5
	12	F/52	NA	NA	6	0	0.3	0.5	IO	1	STR	24
LOA	2	F/59	Ib	24	NA	NA	0	0	IO	4, t, OM	GTR	94
	5	F/54	Ib	40	NA	NA	0	0	IO	5, t, OM	GTR	0
	7	F/40	Ib	29	6	0	0	0	IO	4, t, OM	GTR	0
	16	F/57	Ib	25	8	1	0	0	IO	5, t	GTR	1
	25	F/48	Ib	30	3	0	0.001	0	IO	4	STR	1
	3	M/33	IIIa	26	3	0	0	0	IO + OC + IC	4, t, OM	GTR	0
	1	F/81	NA	NA	9	0	0	0	IO	5, t, OM	GTR	54

*Preop* Preoperative; *Postop* Postoperative; *IO* Intraorbital; *OC* Optic canal; *IC* Intracranial; *OM* Ophthalmectomy; *t* Transection of optic nerve; *EOR* Extent of resection; *GTR* Gross-total resection; *STR* sSubtotal resection

^*^According to Schick et al.

^†^1, endoscopic transnasal approach (ETA); 2, ETA combined with transconjunctival approach (TCA); 3, endoscopic optic nerve decompression (EOND); 4, lateral orbitotomy approach (LOA); 5, LOA combined with TCA

**Fig. 3:**
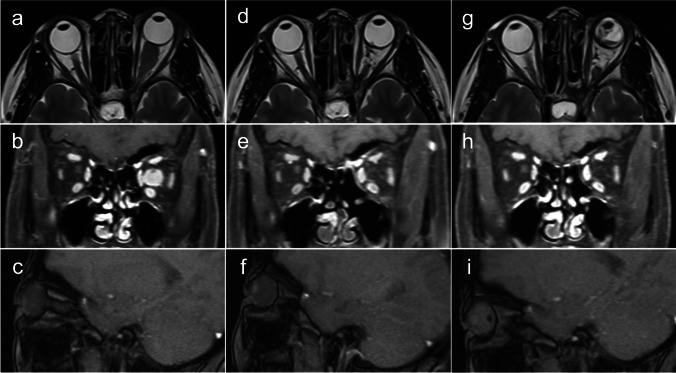
Case 11 presentation. MRI showing a left-sided ONSM underwent ETA. **a**–**c** At 1-day preoperative axial, coronal, and sagittal T1/T2-weighted MR images; **d**–**f** at 6-month postoperative axial, coronal, and sagittal T1/T2-weighted MR images; **g**–**i** at 34-month postoperative axial, coronal, and sagittal T1/T2-weighted MR images

**Fig. 4:**
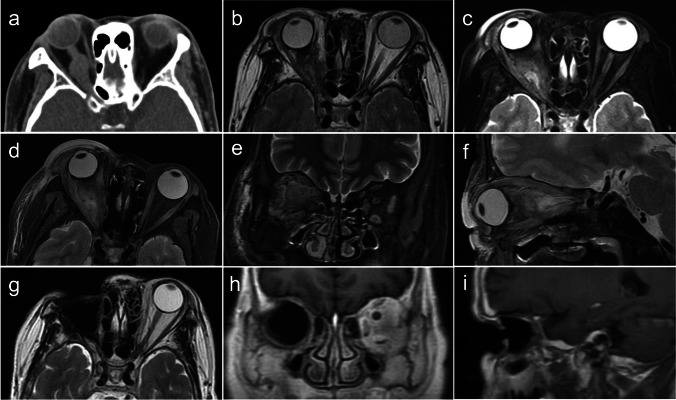
Case 2 presentation. Computed tomographic (CT) and MRI showing a right-sided recurrent ONSM underwent lateral orbitotomy approach (LOA) in April 2014. After LOA, there was no sign of recurrence. **a** CT image in October 2012; **b** axial T2-weighted MR image in January 2013; **c** axial T2-weighted MR image with Gd enhancement in February 2014; **d**–**f** axial, coronal, and sagittal T2-weighted MR images in April 2014; **g**–**i** at 94-month postoperative axial, coronal, and sagittal T1/T2-weighted MR images

In the ETA group, there were 16 cases, of which one case had light perception (LP) and three cases had no light perception (NLP). Among the remaining 12 cases, four cases had poor vision, seven cases had fair vision, and one case had good vision. GTR was achieved with ONT in 14 out of 16 (87.5%) patients (Fig. 3). Despite the loss of vision, the normal movement function of the eye was still retained, as observed from appearances before the operation. OM was necessary for one patient due to tumor recurrence and poor ocular condition. The predominant complications observed were intraoperative cerebrospinal fluid (CSF) leakage (three out of 16 cases) and transient oculomotor nerve palsy (four out of 16 cases). CSF leakage resolved completely following strict bed rest. Oculomotor nerve palsy also resolved within 2 months postsurgery. Some temporary reduction in extraocular motility and no ptosis were observed postoperatively. Although some patients reported occasional symptoms of eye pain or fatigue after the surgery, preexisting dizziness and headache resolved in these patients after surgery. In the ETA group, the average follow-up duration was 17.38 months and there were no patients who experienced recurrence or intracranial expansion (Fig. 3). In the LOA group, six out of seven patients had NLP, one patient had LP, and two others had optic atrophy. Tumors had arisen de novo in three cases, all of which recurred in the orbit after previous treatment with intraorbital tumor resection several years prior. GTR was achieved in six out of seven (85.71%) patients (Fig. 4), and STR was achieved in one patient (14.29%). The extent of tumor resection achieved by both methods was similar, but cosmetic results differed. Most patients in the LOA group achieved GTR through OM. However, during a telephone interview, patients complained that the disfigurement caused by the lack of eyeball seriously affected their social life and reported that even with artificial eyes, they could not achieve a natural appearance and that ocular prostheses would interfere with daily exercise. There was no postoperative infection or recurrence (Figs. 3 and 4). The length of hospitalization did not differ significantly between the two procedures (*p* = 0.1721, *t* = 1.414).

### Illustrative case 27

**Fig. 5:**
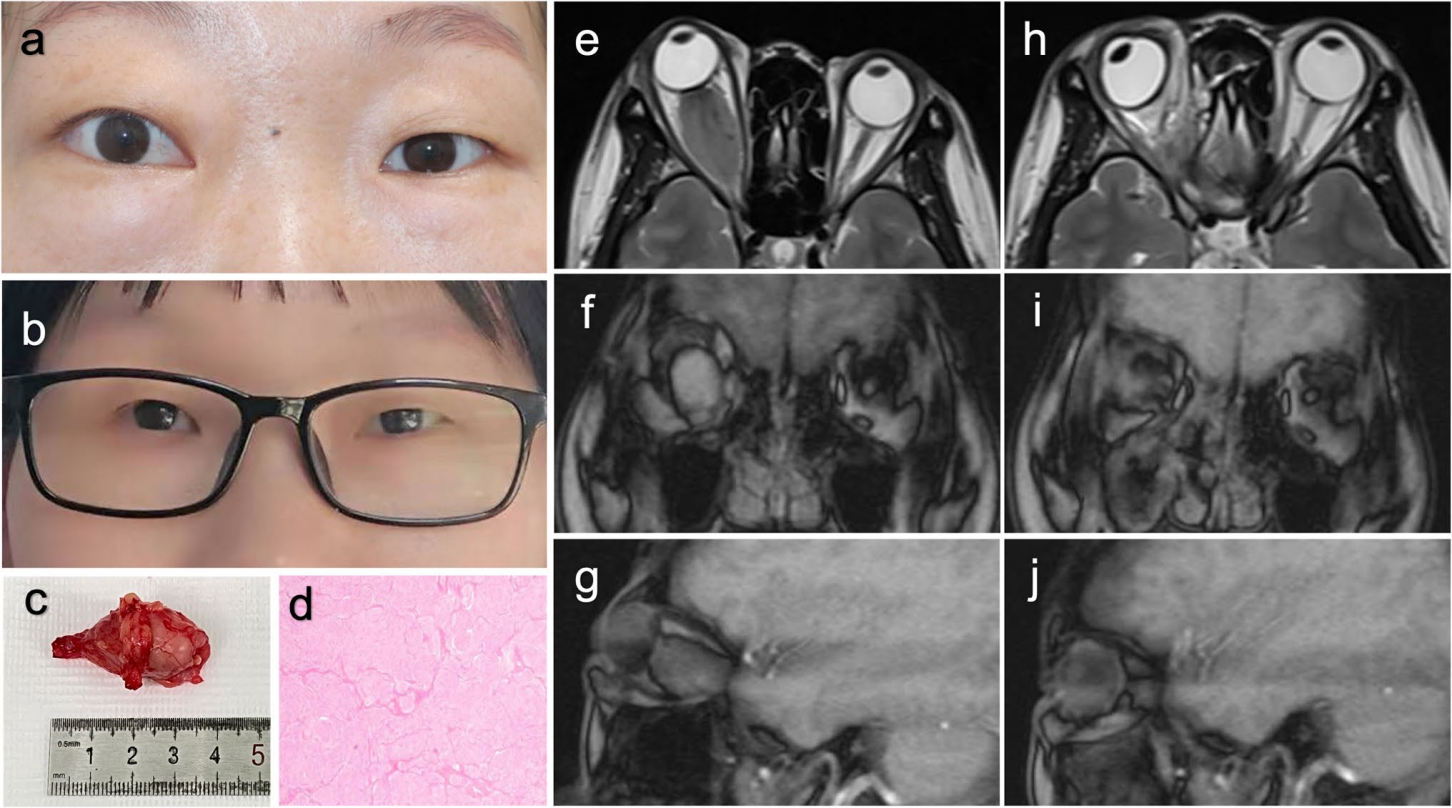
Case 27 presentation. **a**,** b** Preoperative and postoperative appearance at the 30-month follow-up; **c** the picture of the tumor just removed; **d** hematoxylin–eosin (HE) staining of the mass; **e**–**g** at 2-week preoperative axial, coronal, and sagittal T1/T2-weighted MR images; **h**–**j** at 4-week postoperative axial, coronal, and sagittal T1/T2-weighted MR images

A 36-year-old female presented with a 36-month history of exophthalmos (9 mm) and a 1-month history of visual impairment in her right eye. Preoperatively, evident exophthalmos was observed (Fig. 5a). A postoperative self-portrait (Fig. 5b), taken during the 30-month follow-up, depicted no significant exophthalmos. The resected tissue is illustrated in Fig. 5c, revealing tumor encasement of her right optic nerve. Pathological analysis confirmed the tumor as an optic nerve sheath meningioma (Fig. 5d). MRI images revealed a round lesion involving the full length of the optic nerve in her right orbit (Fig. 5e–g), with dimensions of 17 × 34 × 20 mm. Total tumor removal was successfully achieved using the ETA. Postoperative images are presented in Fig. 5h–j, with no immediate complications observed. Telephone follow-ups at 9 and 30 months postoperation indicated the patient’s return to normal work and life, with a positive mood and absence of related complications.

## Discussion

Despite the relatively benign behavior of ONSMs, surgical excision is often warranted to prevent extension, particularly when accompanied by disfiguring proptosis or severe ipsilateral visual loss. Unfortunately, the location of these lesions relative to the optic nerve often results in high visual impairment associated with surgical intervention, with over 90% of cases resulting in blindness following complete resection [11]. Controversies in the surgical management of ONSMs have existed over the last two decades [2]. In this paper, we describe a novel surgical technique and suggest some preoperative indications of ETA with ONT for patients who had the intention of total tumor resection. By highlighting our clinical experience utilizing this technique, we strive to demonstrate that this less invasive approach offers satisfactory access to the apex of the visual cone without resorting to conventional craniofacial approaches, which may be complicated by facial incisions and brain retraction, or lateral orbitotomy approach with OM, which may evoke disfigurement and mental distress. Ultimately, our study suggests that this microinvasive technique leads to noteworthy cosmetic outcomes and holds the potential for achieving radical cures of ONSMs.

Although the main treatment strategy concerning achieving the best vision enhancement outcome is essential to choose the most appropriate approach, safe and effective prevention of further tumor damage to the opposite eye or brain becomes a more important consideration when the patient experienced long-term vision loss and/or disfiguring proptosis. Peerooz Saeed et al. suggested that patients with a blind eye and significant proptosis were candidates for total excision [1]. According to the tumor location of 73 patients with ONSMs, Schick et al. classified ONSMs into different types and subtypes. Among those, nine patients with preoperative blindness underwent transection of the optic nerve [10]. In another study reported by them, 11 blind patients with painful disfiguring proptosis, whose ON was crossed [12]. In a series of 24 cases of ONSM, seven cases with blindness or light perception performed an ONT and none of them had either clinical or radiological recurrence [13]. In four of our 23 cases, they all recurred in orbit due to non-total resection of tumors. And whether underwent ETA or LOA, after GTR with transection of the ON, none of them recurred either (Figs. 2 and 3). To sum up, complete surgical resection with ONT is rarely warranted in the management of ONSM except in patients suffering from blindness or disfiguring proptosis. Transaction may also have a role if the tumors with the tendency of intracranial extension, to prevent chiasmatic spread to the contralateral ON [2, 14]. Therefore, it is virtually possible to extirpate these tumors and reduce the risk of recurrence. How to transect the optic nerve without compromising the surrounding normal neurovascular structures to complete tumor resection is an important problem. The endoscopic transnasal approach may be the best answer.

Significant advances in endoscopic transnasal (ETA) and microinvasive surgical approaches have revolutionized the intervention of orbital tumors. And ETA has a wide range of flexible modifications and coordinations available depending on the origin and extent of the targeted disease [15, 16]. In previous studies, endoscopic techniques were introduced to bony decompress the orbital tumors [17, 18] and dramatically improved the outcomes in skull base tumors surgery such as sellar [19–21], olfactory groove [22], and spheno-orbital meningiomas [23]. Nevertheless, the application of ETA in meningiomas involved in the optic nerve is rare. Four primary ONSMs cases were treated with EOND surgery with stable clinical results [24]. Endoscopic transnasal surgery was performed for one case of aggressive optic sheath meningioma. Partial removal of the tumor was achieved and visual symptoms of the patient were significantly improved [25]. Similarly, in another patient with long-lasting blindness, the fully endoscopic technique enabled surgeons to subtotal remove the lesion [26].

In our series, three cases with NLP, one case with LP, and 10 cases with residual vision underwent ETA and ONT joint surgery. According to the recommendations of treatment for different tumor types from Schick et al., all patients opted for surgery in compliance with the recommendations [10]. Based on previous research, only patients with bad visual acuity and disfiguring proptosis were subject to transection of the ON, but our data concerning the subset of patients who had vision greater than NLP or LP at presentation are included. For these patients, there are four main reasons for GTR with ONT: (1) patients and their families were more concerned about the mass than vision loss; (2) the mass increases and affects vision gradually; (3) there is a risk of intracranial metastasis; and (4) exophthalmos affect patients’ appearances. Visual loss after transection surgery is the norm, and reasonably, tumors with greater tightness with the optic nerve have less chance of attaining visual cure under micromanipulation. In the absence of a more ideal management algorithm, ETA performed in these patients is still an available and microinvasive option, especially in adults with unstable vision and disfiguring proptosis. On the other hand, ONT of symptomatic patients could preclude contralateral visual injury.

In our view, the risk of affection of the contralateral optic nerve and brain and a large volume of tumor are also indications of ONT, not just vision loss and/or disfiguring proptosis. Even though there are optimal approaches in a minimally invasive 360° circumferential access to target orbital tumors to avoid crossing the plane of the optic nerve [4, 5], as a tumor arising from the arachnoid of the intraorbital optic nerve, ONSM is difficult to be removed completely without damaging the optic nerve. The cavernous hemangioma exclusively endonasal resection (CHEER) staging system was developed in 2019 to facilitate research on endonasal resection of orbital cavernous hemangiomas [27]. And direct retraction of the optic nerve was avoided in this staging system. The more locations where tumors clearly involved, the higher stage was used. Because the inferomedial muscular trunk of the ophthalmic artery (IMT) is considered to be the next most important intraorbital structure following the ON [28], the orbital space posterior to the IMT (stage IV) is divided into a high-risk zone where the lesion lie within millimeters of the ON. A meta-analysis suggested that the CHEER staging system had a broader application to benign orbital tumors including meningioma [29]. Based on this staging system, there are 11 lesions anterior to the optic canal (stage IVA), four lesions extending through the optic canal (stage IVB), and one lesion extending into the intracranial location (stage VA) of our 16 cases in ETA group. Taking the opinions of multidisciplinary panelists in orbital tumor surgery into account, the expectation of complete excision by endonasal resection declined with higher stages (stages IV and V) while the anticipated risks for vision loss increased [27]. Therefore, it was very difficult for our patients to remove the tumor without ONT. Even though the Simpson Grade may be antiquated and hard to predict recurrence-free survival for meningiomas according to recent studies [30–34], the goal of surgery should also be the maximal safe resection of the tumor. And combined with ONT, GTR can be obtained more safely and efficiently.

By follow-up via telephone, all patients who received ONT said they preferred complete resection of the tumor knowing they had a tumor and would lose vision. And for them, a minimally invasive approach that preserves the eyeball was better. Case 18 clearly stated that she became unconfident and self-abasement due to changes in her appearance after the OM. Although most patients undergoing OM were lost to follow-up due to age or the long length of the operation, the psychological problems of patient postoperation could not be neglected. Increasing attention is diverted to studying dimensions of preoperative and postoperative quality of life (QOL) of patients. In this regard, however, there have been so many methodological investigations of the improvement of vision of patients with ONSMs, omitting a psychopathological snake in the grass.

In comparison to intracranial tumors [35–37], none of foray has been made to quantify mental health for patients with tumors involved in the optic nerve because of low incidence, slow progression, and good prognosis. Our experience suggests that ONT is not only a favorable result for blind patients, but also an early intervention, a less mental burden, and a certain predictability for an individual with/without fair or good visual performance who is faced with an upcoming blindness and larger tumor mass. Therefore, it is unnecessary to just wait until the patients’ conditions meet the traditional indication to have an ONT. Although many treatment options can preserve the integrity of the optic nerve and improve vision, the associated risks of complications and recurrence should not be ignored [38]. A meningioma-specific quality-of-life questionnaire was designed specifically for evaluating QOL in meningioma patients [39]. And we tried to perform interviews via telephone with patients to better understand and capture health-related QOL in the ONSM population. Unfortunately, due to the low education level of the patients and the fact that more than half of the patients were over 50 years old, it was difficult to communicate accurately and fully assess their postoperative QOL. Also, it was unfortunate that patient-reported QOL in endoscopically managed orbital meningioma has never been well elucidated, nor has the surgical effect on QOL been ascertained as evidence is limited to small cohorts. We hope that with larger, multicenter collaborative studies in the future, the role of endoscopic transnasal approaches for QOL in ONSM management will become clearer. Thus, there can be more evidence to break the rules and broaden the applicability of ONT.

In the LOA group, poor eyesight of patients led to the justifiability of ONT. Unfortunately, the only downside was the removal of the eyeball, which could diminish patients’ quality of life due to the lack of natural appearance and limited movement of artificial eyeballs. Though no professional questionnaire was used to evaluate this conclusion, OM during surgery should be avoided. Compared to the two groups of clinical outcomes, without removal of the eyeball and skin incision, ETA provides a shorter and more direct operative corridor to total remove the lesions located in the orbit and optic canal via the transethmoidal-sphenoidal incision. This access-related advantage is clinically associated with excellent cosmesis, reduced postoperative pain, and wound infection. Although ETA with a risk of neurological complications, the defects cannot obscure the virtues. Statistically, the hospitalization time for patients who underwent tumor resection via the ETAs or lateral orbitotomy method both showed a short length of hospitalization (*P* > 0.05). However, LOA obtained GTR by OM, which resulted in poor cosmetic results. The simple LOA method, while preserving the eyeball, can also leave surgical incision scars on the face, thereby affecting aesthetics. It is therefore evident that ETA serves as a microinvasive approach that is associated with excellent cosmetic outcomes and may be better suited for patients seeking a minimally invasive procedure. However, all the LOA selected in this study were performed before 2017. Advancements in contemporary techniques suggest that such drastic measures may not be universally required, and preservation of the eye and orbit can be achieved. Additionally, all the ETA procedures selected in this study were performed after 2016. It is noteworthy that potential bias was introduced when comparing LOA to ETA. This study needs further refinement of the techniques for both surgical methods in the future. While ensuring the preservation of the eyeball in both approaches, it is essential to clarify the differences in patient cosmetic outcomes resulting from these two surgical methods. Moreover, there is a learning curve for surgeons to proficiently perform ETA. Also, during ETA, particular emphasis should be placed on safeguarding the extraocular muscles while approaching the optic nerve mass. Extraocular muscle injury or vascular bleeding can occur easily, especially in inexperienced hands. Therefore, patient preference and the surgeon’s extensive surgical experience are particularly crucial for selecting such procedures.

## Conclusions

While we acknowledge that the aim of surgery is usually to achieve better control of visual outcomes, ONT combined with ETA may be a suitable option in selected cases to avoid a period of accelerated tumor growth, deterioration, recurrence, and emotional burden. However, larger studies with longer follow-up periods are required to provide further support for this proposal. Nonetheless, ETA combined with ONT offers the benefit of comprehensive symptom relief while preventing disease progression, without resorting to more invasive interventions. Nevertheless, it remains essential to effectively communicate with patients about the potential effects of this procedure on vision and other treatment alternatives, enabling timely intervention for the tumor.

### Supplementary Information

Below is the link to the electronic supplementary material.

Supplementary file1 (MP4 60083 KB)
